# The Use of Medical Hypnosis to Prevent and Treat Acute and Chronic Pain: A Systematic Review and Meta-Analysis

**DOI:** 10.3390/jcm14134661

**Published:** 2025-07-01

**Authors:** Adina Yerzhan, Akbota Ayazbekova, Danielle R. Lavage, Jacques E. Chelly

**Affiliations:** 1 Department of Anesthesiology and Perioperative Medicine, University of Pittsburgh, Pittsburgh, PA 15261, USA; yerzhana@upmc.edu (A.Y.); ayazbekovaa@upmc.edu (A.A.);; 2 Center for Complimentary Medicine, University of Pittsburgh, Pittsburgh, PA 15232, USA

**Keywords:** hypnosis, medical hypnosis, pain scores, analgesia, acute pain, chronic pain, alternative pain management

## Abstract

**Background/Objectives**: In the current opioid crisis, medical hypnosis has been proposed as an alternative to opioids to control acute and chronic pain. The aim of this study was to use a meta-analysis to conduct an objective assessment of the value of medical hypnosis for the management of acute and chronic pain and opioid consumption. **Methods**: An initial PubMed search showed 111 relevant studies out of 1115. Twelve randomized controlled studies (RCTs) were identified, published from January 2014 to December 2024, focusing on acute and chronic pain. These RCTs were analyzed to compare the effects of medical hypnosis vs. standard care. **Results**: The use of medical hypnosis for acute pain was found to decrease pain by 0.54 standard deviations (SD) compared to the standard care, and the effect was medium and statistically significant (95% CI [0.19–0.90]; *p* = 0.0024). Oral morphine equivalents (OME) in the hypnosis group were 1.5 SD lower than in the placebo group, which was statistically significant (95% CI [0.12, 2.88]; *p* = 0.03). Unlike the effectiveness of hypnosis for acute pain treatment, hypnosis was not found to have any impact on the treatment of chronic pain. The mean pain value difference in the hypnosis group had little effect and showed a statistically insignificant result—a Hedges’ g score of 0.07 (95% CI [−0.14–0.27]; *p* = 0.518). **Conclusions**: The use of medical hypnosis was associated with a statistically significant decrease in acute pain scores and OME, suggesting it is a potential alternative to opioids, but our analysis indicates that hypnosis does not reduce chronic pain.

## 1. Introduction

Hypnosis is a complementary technique based on focus, attention, relaxation, and the alteration of perception, allowing for changes in the behaviors controlled by the subconscious. Priests and healers in ancient Greece, Egypt, China, India, and even Europe began using this technique centuries ago to treat different conditions using mystical and religious rituals [1]. There is evidence to support that as early as the third century before the Common Era, Egyptians practiced healing in a “healing temple” based on first having the patient pray and then placing the patient in a dark room where the patient was expected to fall asleep. This sequence of practices, in combination with direct interaction with a priest, was the basis of the healing. Similar practices were observed in Greece, where symbolic dreams were interpreted and altered by priests to promote recovery. In some cases, the dreams themselves were considered to have therapeutic effects [2].

Evidence indicates that the medical use of hypnosis started in the 18th century [1]. The earliest evidence of the use of medical hypnosis appeared around 1766 in Vienna, when Franz Anton Mesmer proposed the theory of “animal magnetism.” Mesmer believed in a bodily fluid that connected humans with the planets and claimed he could heal diseases by redistributing this magnetic fluid to restore equilibrium [3]. Although this theory was discredited in 1787 when controlled experiments with blindfolded patients demonstrated that it was the power of imagination rather than magnetic fluid at work, public interest in mesmerism persisted. In the 19th century, James Braid introduced the term “hypnosis,” coming from the Greek word “hypnos,” meaning “sleep.” Hypnosis was first used for surgical anesthesia in 1829 by French surgeon Jules Cloquet for breast tumor removal. In the post-operative period, patients did not remember anything about the surgery. Later, in 1836, animal magnetism was successfully used to reduce pain for dental extraction in the same manner [4]. The practice was later expanded by John Elliotson, who published 76 case results demonstrating the use of hypnosis as a sole anesthetic. This practice continued until the introduction of chloroform in 1847 [5].

In the modern era, hypnosis can be used for the treatment of acute and chronic pain, and a review by Patterson et al. and a meta-analysis by Thompson et al. demonstrated that hypnosis has consistently demonstrated superiority compared to standard care in randomized clinical studies [6,7]. Medical hypnosis, alone or in combination with cognitive behavioral therapy, has shown promising results as a therapeutic technique for the control of anxiety, depression, smoking cessation, weight loss, and inflammatory bowel disease [8]. Moreover, the use of hypnosis has been shown to enhance post-operative recovery, to shorten the length of hospital stay, and to reduce the use of opioid and non-opioid medications [8,9,10,11].

Hypnosis comprises three main phases. The first phase, the induction phase, starts with asking the patient to progressively or suddenly focus on a single stimulus, image, idea, feeling, or behavior, like breathing or sleeping. This phase is usually achieved through the hypnotist’s guidance to induce the patient to relax and enter a “trance” or alter the patient’s level of consciousness. After the patient reaches this stage, the next phase, the maintenance and deepening phase, can begin. This phase is understood to allow the patient to reach a deeper focus, depending on the patient’s sensitivity to a “suggestion” or external command. The last phase is the recovery phase. This phase allows the patient to recover consciousness without memory of the suggestions or commands given during the maintenance phase. Patients are not equally susceptible to hypnosis [12]. The degree to which every patient can be hypnotized can be assessed using validated instruments such as the Stanford Hypnotic Clinic Scale [13].

Induction methods vary in several ways: they can have either an authoritarian or a permissive tone, require a relaxed or alert–awake state, use suggestions that are either lengthy or brief, and be either difficult or easy to follow. Nonetheless, patient responsiveness does not seem to vary widely across different protocols [14]. One way to categorize them was suggested by Kroger, who emphasized that the cumulative effect of hypnosis requires an effectively initiated and reinforced monotone, repetitive approach, free from distractions. He described the following three main types of induction:

- Direct techniques: These practices correspond to the eye fixation method, where the patient focuses on an image, leading to muscle fatigue and relaxation, which helps achieve hypnosis.
- Indirect techniques: These methods are based on the use of visual imagery, such as asking the patient to imagine a moving screen in their mind to facilitate the hypnotic state.
- Mechanical techniques: These practices utilize repetitive mechanical, visual, or tactile stimuli, often involving tools like metronomes or rhythmic electronic instruments.

These techniques have been shown to generate slow alpha waves in the brain, promoting drowsiness [15]. While induction is commonly seen as a tool to initiate hypnotherapy, the Ericksonian approach integrates induction as part of the therapeutic process, using conversational techniques, metaphors, and indirect suggestions rather than direct commands from the hypnotist [16,17]. The Ericksonian approach integrates a suggestive tone and uses everyday life to work towards the realization of a desired goal rather than dissociation.

The maintenance and deepening phase involve helping the patient to deepen their level of unconsciousness. It is usually undertaken to reach a higher level of relaxation and/or focus by asking the patient to feel more and more sleepy and focus on an object, situation, etc., by integrating therapeutic suggestions. To achieve therapeutic goals, the method used to deliver suggestions is less important than the patient’s openness, readiness, attitude, expectations, and ability to respond to suggestions [14]. Nonetheless, it is also important to consider individualized approaches based on subject characteristics and the hypnotist’s abilities.

The exact mechanism by which hypnosis affects neurophysiology has not yet been determined. In the case of its effect on pain, it is proposed that hypnosis produces a dissociation of the awareness of painful stimuli from the actual sensory experience, allowing for pain to be present but not perceived [18]. The most common animal model for testing hypnosis-like states is the rabbit, specifically through the tonic immobility state (TIS), which is considered similar to hypnosis in humans. Castiglioni et al. demonstrated that TIS rabbits’ reaction to pain stimuli was similar to the analgesic effects of lidocaine [18].

Overall, the goal of this review was to assess the correlation between medical hypnosis and acute perioperative pain, chronic pain, and opioid requirement using a meta-analysis.

## 2. Materials and Methods

### 2.1. Eligibility Criteria

We began by searching PubMed for articles related to hypnosis, acute pain, and chronic pain. We then selected studies that included randomized controlled trials (RCTs) conducted in adult humans. These studies compared the effects of hypnosis—administered pre-operatively, intraoperatively, or post-operatively—to standard care, using a numerical rating scale (NRS) for pain (0 = no pain to 10 = worst possible pain) to assess both acute and chronic pain. We accepted both direct and indirect methods based on the use of pre-recorded audio. Studies that did not report on the association between pain score and hypnosis, did not use NRS values for these associations, or that included pediatric patients, as well as observational studies, review articles, meta-analyses, letters, and abstracts, were excluded.

### 2.2. Search Strategy

PubMed search engines were used to retrieve studies including the terms “hypnosis” AND “acute pain” and “hypnosis” AND “chronic pain” and “hypnosis”. We selected articles published between January 2014 and December 2024 for acute and chronic pain. No additional search limitations were used. Two independent researchers assessed trials, and the list was retrieved in December 2024.

### 2.3. Data Extraction

For each RCT, the number of patients in each study group, the study duration, a description of the hypnosis treatment, the type of hypnosis induction, the mode and frequency, the type of surgery, and the type of pain being treated (acute or chronic) were collected. The primary outcomes were pre-operative and post-operative pain scores for acute pain and pre- and post-treatment scores for chronic pain assessed by NRS and/or the visual analogue scale (VAS) using a scale of 0 = no pain to 10 = worst possible pain. For acute pain, the meta-analysis was first conducted using combined data for both scores. Because heterogeneity scores differed between the NRS and VAS measures, we performed a secondary analysis separately for each scale. Since pain was reported at different times according to each study, data reported at baseline and at post-treatment hour 24 were included. The secondary outcome was average oral morphine equivalents (OME) for acute pain. The same average pooled OME analysis was not conducted for chronic pain papers due to the limited number of reported OMEs in the included studies. The risk of bias was assessed using the Cochrane risk-of-bias tool for randomized trials (RoB 2), and visualization was conducted via robvis [19].

### 2.4. Statistical Analysis

The mean differences with standard deviation (SD) of the pain scores and OME (mg) were reported for comparison between the hypnosis treatment and control groups. In the studies containing several comparison groups, only hypnosis treatment and control groups were taken into consideration. Analyses were calculated using a random effect model because of clinical heterogeneity. Heterogeneity was assessed based on I^2^ statistic scores. Heterogeneity was considered low if I^2^ scores were lower than 25%, medium if I^2^ scores were lower than 50%, and high if I^2^ scores were above 50%. A forest plot was built for the assessment of the risk of bias publication using Hedge’s g score, which was calculated to evaluate effect size as some of the studies contained a small number of subjects. The magnitude of difference was interpreted as small when Hedge’s g was around or less than 0.2, medium when Hedge’s g was around g = 0.5, and large when Hedge’s g was around or above 0.8. A 95% confidence interval (CI) was used to report the differences between groups. Sensitivity and sub group analysis was performed to determine the cause of possible heterogeneity. The meta-analysis was performed using Stata 18 software.

## 3. Results

### 3.1. Acute Pain

**Figure 1:**
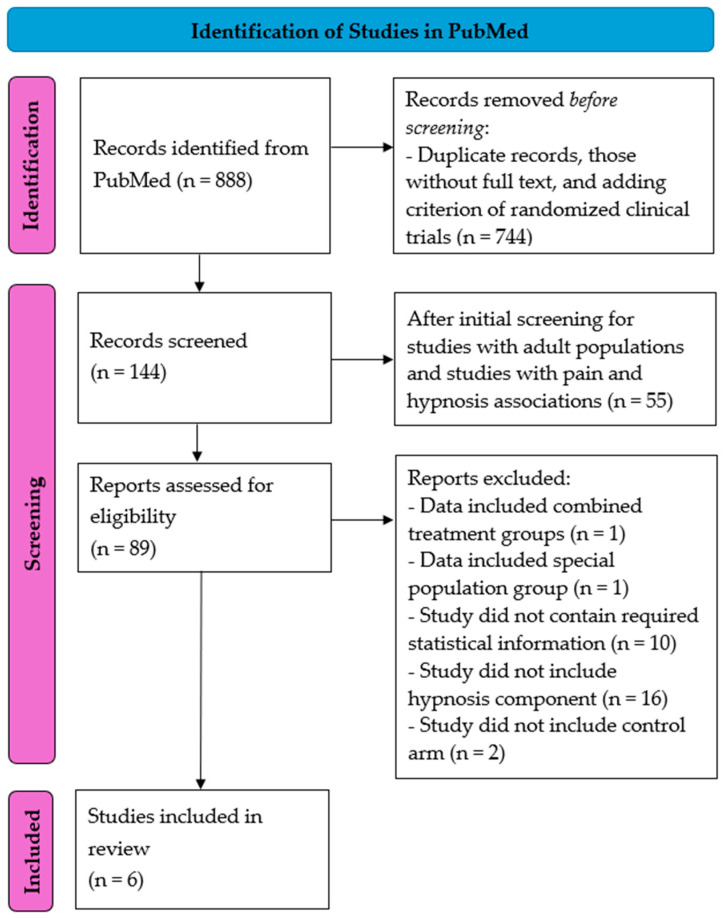
PRISMA chart showing the selection process for studies that described the association between acute pain and hypnosis treatment for meta-analysis.

Based on our literature search, 888 study results emerged. After removing duplicate records and those without full text and adding the criterion of RCTs, 144 studies remained. Furthermore, after selecting the results based on the inclusion and exclusion criteria, 89 manuscripts remained. The study selection PRISMA chart is shown in Figure 1.

Overall, six studies included in the meta-analysis were RCTs [20,21,22,23,24,25]. The studies involved a total of 888 patients, ranging from 17 to 385 per study. For each study, the nature of the effect (medium or high) and the heterogeneity, I2, were established. These studies included different types of pain stimuli, including peripheral intravenous cannulation, biopsy, and lung transplantation. The hypnosis modes were mainly indirect, employing pre-recorded audio suggesting relaxation and calmness. One study a used direct induction mode, whereby the hypnotherapist used eye-fixation methods [22]. The control groups were exposed only to standard care. Each study’s characteristics are shown in Table 1. Pain scores were expressed as either NRS or VAS scores. A description of the hypnosis treatment, frequency, results, and number of participants in each arm is also included in Table 1.

#### 3.1.1. Primary Outcome: Acute Pain Scores

Of the six studies, three used the NRS and three used the VAS to assess pain [19,20,21,22,23,24]. Table 2 depicts the mean values, SDs, and the number of patients in each study depending on the type of score reported for pain perception.

When analyzing the selected papers individually, all studies except for those by Lee et al., 2019. and Amraoui et al., 2018. showed statistically significant pain reduction in the hypnosis group compared to the placebo group [23,24].

**Figure 2:**
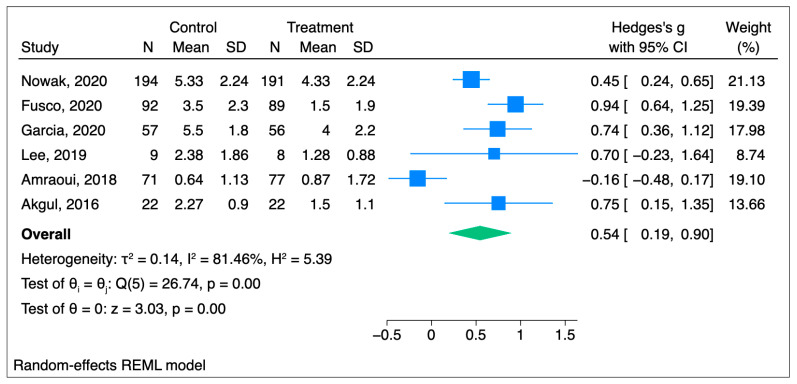
Forest plot for acute pain analysis [20,21,22,23,24,25].

The mean pain value in the hypnosis group is 0.54 SD lower than that in the placebo group; the difference has a medium effect and is statistically significant (95% CI [0.19–0.90], *p* = 0.0024). Hedge’s g score for the study by Amraoui et al., 2018, was negative [24]. The pain score in Amraoui et al. was higher in the hypnosis group than in the control/placebo group. All other studies demonstrated the effectiveness of hypnosis for acute pain treatment. Heterogeneity was found to be high; I^2^ = 81.46%, as depicted in Figure 2.

#### 3.1.2. Secondary Outcome: Oral Morphine Equivalents

Among the studies, three described opioid consumption, along with OME (mg), as shown in Table 3.

**Figure 3:**
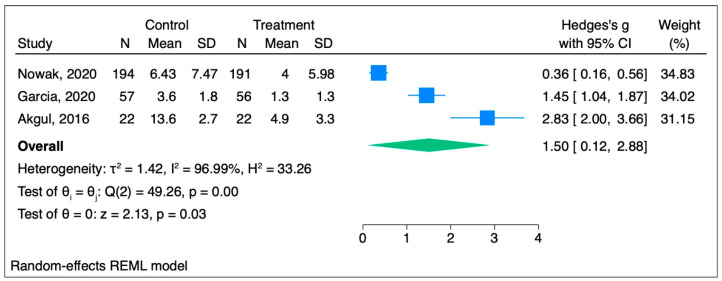
Forest plot for opioid consumption analysis [20,22,25].

OME in the hypnosis group was 1.5 mg SDs lower than in that in the placebo group, and the difference had a significant effect as the Hedge’s g score was more than 0.8. The forest plot in Figure 3 shows that the difference is statistically significant (95% CI [0.12,2.88]; *p* = 0.03).

#### 3.1.3. Subgroup Analysis

**Figure 4:**
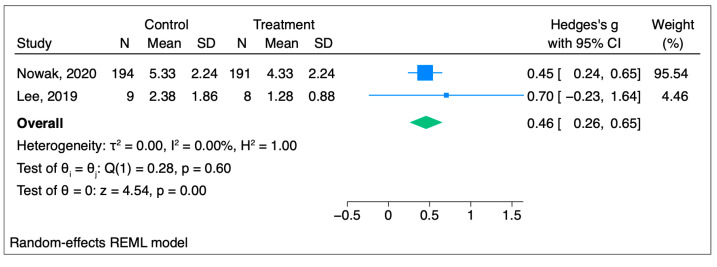
Forest plot for audio hypnosis group [20,23].

Subgroup analysis was performed, and we found that in both the audio hypnosis and live hypnosis groups, there was a statistically significant reduction in the pain score and that the reduction is lower in the live hypnosis group than in the audio hypnosis group, leading to the hypothesis that live hypnosis could be more efficient than pre-recorded hypnosis sessions. The forest plot in Figure 4 shows that in two studies that used audio, Hedges g’s score was 0.46 [95%CI 0.07–0.82] (*p* = 0.02), which shows a statistically significant reduction in the pain score in the hypnosis group [20,21,22,23].

**Figure 5:**
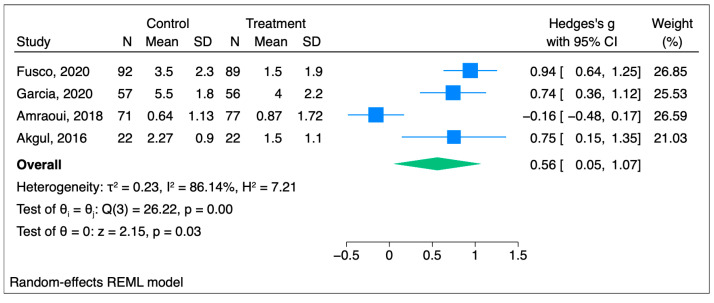
Forest plot for live hypnosis group [21,22,24,25].

For the remaining four papers that used live hypnosis sessions, the Hedges g’s score was 0.56 [95%CI 0.05–1.07] (*p* = 0.03), which shows a statistically significant reduction in the pain score in the hypnosis group (Figure 5) [21,22,24,25].

#### 3.1.4. Sensitivity Analysis

**Figure 6:**
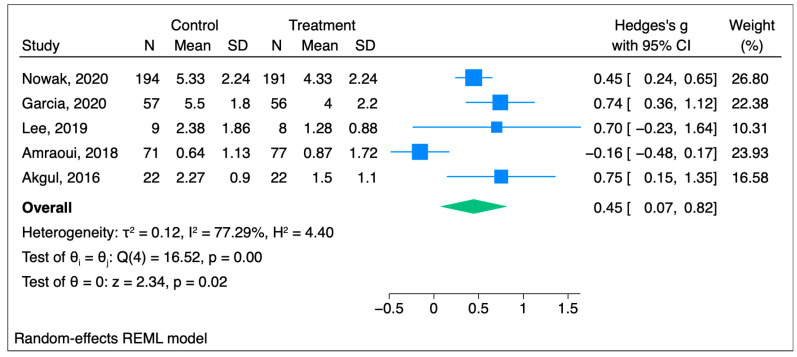
Forest plot for the sensitivity analysis [20,22,23,24,25].

In the acute pain studies, the high degree of heterogenicity in the I^2^ values could be due to several factors, such as the different types of invasive procedures performed (varying from major surgeries like CABG to invasive procedures like peripheral i.v. cannulation (PIVC)), the different degrees of surgical complexity, and the different cohorts of patients with different comorbidities and complications. Moreover, the lack of a standardized consensus hypnosis protocol allows for different hypnotist specialists to perform ithypnosis differently (i.e., live vs. audio, and even among these the two audio studies, one used background music while the second one did not [20,23]). In the sensitivity analysis, where the PIVC cannulation pain measuring paper was removed, in the five remaining studies analyzed, the Hedges’ g score was 0.45 [95%CI 0.07–0.82] (*p* = 0.02 and I^2^ = 77.29%) (Figure 6), which still indicates a statistically significant decrease in the pain score in the hypnosis group and a lower heterogenicity score [21]. It did lower heterogenicity score, even though, albeit not substantially so.

We believe that controlling for different variables could lead to a lower I^2^. However, both extensive sensitivity and subgroup analyses are challenging to perform with this meta-analysis due to the already-small sample size and paper count.

### 3.2. Chronic Pain

**Figure 7:**
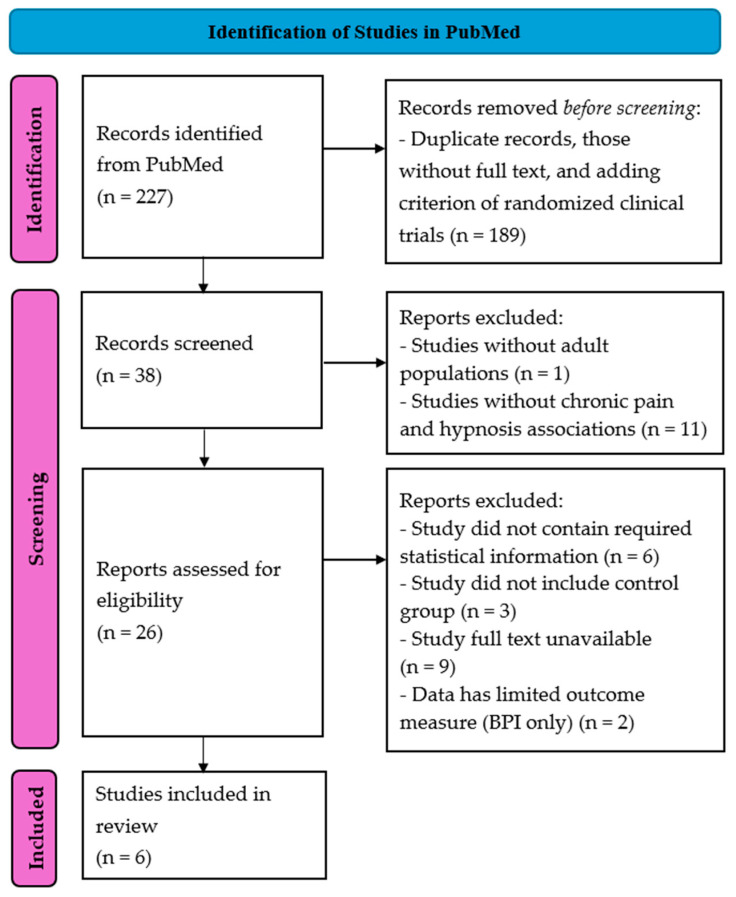
PRISMA chart showing the selection process for studies that described an association between chronic pain and hypnosis treatment for meta-analysis.

Based on the literature search, 227 study results emerged. After removing duplicate records and publications without full text and adding the criterion for RCTs, 38 studies remained. After selecting the results in accordance with the inclusion criteria and exclusion criteria, six manuscripts remained. The study selection flowchart is shown in Figure 7.

The six studies included in the meta-analysis were RCTs [26,27,28,29,30,31]. The studies involved a total of 595 patients, ranging from 20 to 220 per study. The pain exposures studied included pain experienced through chronic diseases, such as joint pain, lower back pain, cancer-related pain, or general chronic pain. The hypnosis modes were indirect, employing pre-recorded audio intended to induce relaxation and calmness. The control groups were exposed to either standard care or pain education lectures. Each study’s characteristics are shown in Table 4.

A summary of the statistical results for mean pain scores after treatment is shown in Table 5.

#### Primary Outcome: Chronic Pain Scores

**Figure 8:**
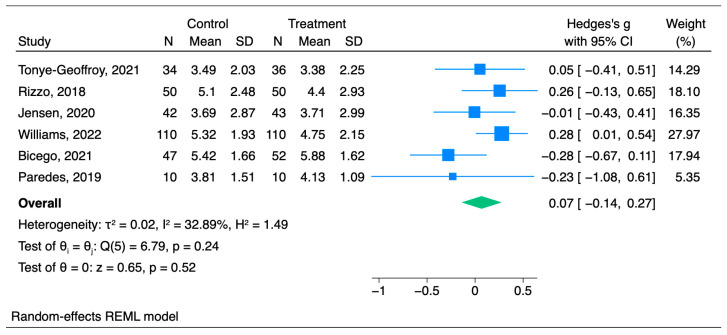
Forest plot for chronic pain analysis [26,27,28,29,30,31].

Unlike the effectiveness of hypnosis for acute pain treatment, hypnosis was not found to have any impact on chronic pain. The mean pain value in the hypnosis group was 0.07 SDs lower than that in the placebo group (95% CI [−0.14–0.27], *p* = 0.518), but as *p* > 0.05, the results are statistically insignificant. Hedge’s g scores for scores for 4 studies were negative. The pain scores were higher in the hypnosis treatment group than in the control group [28,29,30,31]. The forest plot in Figure 8 illustrates that the heterogeneity was found to be I^2^ = 32.89%.

## 4. Discussion

According to a 2017 National Institutes of Health report, the global burden of opioid dependence was estimated at 40.5 million people, and approximately 109,500 people died from overdose [32]. This high rate of opioid dependency has driven the search for alternative pain management strategies, including medical hypnosis, which could either replace opioids or reduce their use. Our data indicates that the use of medical hypnosis may be associated with a reduction in opioid consumption for the control of acute but chronic pain.

Clinically meaningful pain reduction requires at least a two-point reduction in NRS and/or VAS scores, or at least a 30% improvement from baseline [33]. Compared to our findings, Fusco et al., 2020 [21], demonstrated the clinically significant effect of medical hypnosis in a model of mild-to-moderate pain. Milling et al. reviewed 20 years of randomized controlled trials and found a medium effect size for pain reduction across various pain types, and our analysis indicates that for both acute and chronic pain, the benefits do not appear to be clinically significant [34]. Several factors need to be considered: (a) in most cases, the induction techniques used were indirect, using pre-recorded narratives, and delivered by different health professionals, including anesthesiologists and hypnotherapists; (b) there was a high variance in the reported scores, a limited number of included studies, and high I^2^ heterogeneity scores; (c) an overall low mean difference among the two treatment groups was observed. One recent meta-analysis conducted by Thompson et al. suggests that hypnosis has an analgesic effect in healthy individuals with moderate-to-high hypnotic susceptibility when exposed to experimentally induced pain [7]. This finding has also been supported in the context of other invasive procedures, such as tooth extraction, as shown in the analysis by Merz et al. [35]. A meta-analysis by Langlois et al. demonstrated that hypnosis produced a significant moderate-to-large effect size when administered in eight or more sessions for the treatment of chronic musculoskeletal and neuropathic pain, compared to control interventions [36]. Similarly, Jones et al. reported that hypnosis showed an analgesic effect when used as an adjunct to pharmacological treatment or educational components [37]. Our data indicates that that the use of medical hypnosis produced a reduction in acute but not in chronic pain. When combined, these analyses clearly support the effectiveness of medical hypnosis and other complex approaches to pain, accounting for the role played by the placebo effect, the indication, the timing of the treatment, the pain model, and the limited nature of many of the studies conducted.

Mendoza and Capafons suggested that in surgical patients, the perioperative use of hypnosis may be beneficial in reducing the pain and emotional distress associated with procedures and/or surgery. Evidence may also suggest that hypnosis may be effective as a complementary technique for the use of analgesics to reduce pre-operative and post-operative pain, reduce hospital length of stay, and enhance post-operative recovery [8]. Like many complementary techniques, the efficacy of hypnosis is based on the patient’s ability to respond positively to the process. Because patients vary widely in their responsiveness, this variability can introduce bias into studies unless participants are preselected based on their likelihood to respond. For example, in a study on the use of auriculotherapy to treat xerostomia, 66% responded to the treatment [38].

Other complementary techniques have been recommended to control acute and chronic pain and reduce opioid consumption. These include the use of virtual reality (VR), acupuncture, auriculotherapy, relaxation, aromatherapy, and nanotechnology. For instance, in a prospective pilot study by Ganry et al., both VAS stress scores and salivary cortisol levels were significantly reduced after 20 patients used VR to tackle pre-operative anxiety [39]. The positive effect of VR and meditation in decreasing anxiety and depression was also seen in 59 intensive care unit patients [40]. However, a randomized controlled study by Rousseaux et al. showed no statistically significant difference between the use of either VR or hypnosis vs. standard care, suggesting that additional research is required [41]. Acupuncture is one of the ancient adjunct therapies used to reduce pain, stress levels, inflammation, and anxiety. A systematic review by Vicks et al. and He et al. demonstrated its effectiveness on chronic pain and cancer-related pain in comparison with a placebo group, but ambiguous results were also reported. For instance, Giovanardi et al. reported that there was no difference for nonspecific lower back pain when acupuncture was used in combination with standard care rather than standard care alone [42,43,44]. In addition, an RCT by Chelly et al. employed to investigate the efficacy of cryo-auriculotherapy on post-rotator cuff surgery pain and opioid consumption resulted in a 15% decrease in acute pain for up to 14 days [45]. Aromatherapy uses plant-derived volatile chemical compounds that are believed to pass through the olfactory pathway to the limbic system and modulate pain perception. According to Nascimento et al.’s meta-analysis of 35 reviewed studies, aromatherapy aided in a 1.73-point reduction in VAS pain scores for painful stimuli. This is supported by other earlier meta-analyses from Lekhan et al. that showed a statistically significant reduction in pain and inflammation, especially post-operative pain scores when used along with conventional treatment [46,47]. This is also supported by an RCT by Chelly et al., which showed that using lavender and peppermint aromatherapy decreased post-operative pain up to 26% in patients undergoing unilateral total hip replacement surgery [48].

In recent years, experts have advocated for the use of a multimodal approach combining complementary techniques and analgesics to treat acute and chronic pain and reduce opioid requirements. According to Hassan et al., integrative medicine comprising pharmacotherapy and complementary and alternative medicine (CAM) significantly reduces opioid use [49]. However, careful selection of CAM should reflect the patient population and type of pain; for instance, both acupuncture and tai chi seem to be more effective for lower back pain, especially in the elderly population [50]. It is possible that selectivity of patients and conditions also applies to hypnosis and its efficacy in the treatment of acute and chronic pain.

One limitation of the use of hypnosis includes the fact that the technique requires specific training. However, different authors, including Nowak et al., Lee et al., Tonye-Geoffroy et al., Jensen et al., and Williams et al., considered that the use of hypnosis through pre-recorded narratives or by asking non-trained healthcare professional to read a pre-established narrative may represent an effective alternative [20,23,26,28,29]. Our analysis indicates that such an approach has clearly been validated, but it is difficult to promote the use of hypnosis. The limited number of studies and the inconsistency of the results further complicate our efforts to produce an objective assessment. Kekecs et al. found that live presentations were more effective than recordings for post-operative pain, while Montgomery et al. reported no significant difference between live and audiotaped interventions [51,52].

Overall, medical hypnosis demonstrated a statistically significant technique to decrease acute pain scores and OME. However, our data failed to demonstrate its effectiveness in the case of chronic pain. This lack of evidence to support the use of hypnosis to control chronic pain may be due to the fact that more complex mechanisms are involved in the development of chronic pain compared to acute pain, to the difficulty faced in consistently and accurately measuring chronic pain (as it might wax and wane and/or be inconsistent), and to the difficulty faced in administering universal and consistent hypnosis treatment in studies reporting the use of hypnosis in chronic pain [26].

The limitations of this study include the following: the power, which may have been affected by the limited number of articles available to this meta-analysis; the fact that both NRS and VAS were used to quantify pain, hindering a general analysis without stratification; and the use of a wide variety of hypnosis methods. The lack of double blindness might also affect RCT quality; out of the six analyzed papers, only in three studies were the researchers blinded but not the participants [27,28,29]. Other 3 studies were open-label studies [26,30,31]. Similarly, for the acute pain study, only half of the studies were double-blinded RCTs [20,22,25]. Consequently, there was a moderate risk of bias (Supplementary Figure S1) for the acute pain studies and a higher risk of bias for the chronic pain studies, as well as small sample sizes (n = 8–194), all of which could potentially be drawbacks. In addition, the search strategy used the broader terms “acute pain” AND “chronic pain”, which may have limited the scope of identified studies. Although this approach followed a predefined study, future reviews should consider more specified or separate search terms to ensure a more comprehensive search of the literature.

Nonetheless, the promising results for acute pain show that hypnosis is a technique that should be investigated using a proper study design, which should include conducting double-blinded RCTs in a large patient population and controlling for factors like age, surgery type, gender, and hypnosis delivery method. Such studies could help clarify the clinical effectiveness of hypnosis, reveal possible mechanisms by which it influences mood and pain, and support the development of a universally applicable hypnosis approach.

## 5. Conclusions

This systematic review and meta-analysis provides evidence that although the use of medical hypnosis reduces acute perioperative pain and opioid consumption, the use of medical hypnosis does not significantly reduce chronic pain. However, these conclusions should take into consideration the important limitations for the results reported on the effects of hypnosis on both acute and chronic pain, including high heterogeneity across studies, the variability in the methods used to perform hypnosis, and the small sample sizes of most of the studies considered. Therefore, further additional and properly designed studies are needed to determine the role of medical hypnosis in managing both acute and chronic pain and opioid consumption, as well as to help identify the populations and the types of surgery that would benefit most from the use of medical hypnosis, either alone or as part of a multimodal pain management strategy.

## Figures and Tables

**Table 1:** Selected studies’ review for acute pain.

Study	Hypnosis Patients (n)	Control Patients (n)	Hypnosis Description	Pain Measure	Outcomes in Mean (SD) [Range]	Surgery/ Procedure
Nowak et al., 2020 [20]	191	194 (blank tape)	**Length:** 20 min of play with 10 min of silence, repeated throughout the surgery **Induction:** Indirect—audiotape through earphones **Role of hypnotherapist:** Authors developed and recorded the text based on hypnotherapeutic principles **Background music:** Trancemusik CD (Hypnos Verlag, Stuttgart, Germany) **Hypnosis susceptibility:** Five-item modified Harvard group scale	NRS	Pain scores were reduced by an average of 25% in the hypnosis group within 24 h after surgery POH2 Intervention = 2 (1.49) Control = 2.67 (2.24) *p*-value < 0.001 POH24 Intervention = 4.33 (2.24) Control = 5.33 (2.24) *p* < 0.001 OME: Intervention = 4.0 (5.98) Control = 6.43 (7.47)	Elective surgery requiring general anesthesia with a planned duration of 1–3 h
Fusco et al., 2020 [21]	89	92	**Length:** Five minutes once during the procedure **Induction**: Indirect—verbal and nonverbal suggestions **Role of hypnotherapist**: Anesthesiologist or nurse with a diploma in therapeutic and hypnotic communication and at least one year of experience	NRS	Hypnosis group pain was lower compared to the neutral group Hypnosis = 1.5 [1.9]; 0–9 Neutral = 3.5 [2.3]; 0–9 *p* < 0.0001 OME: not reported	20G peripheral intravenous cannulation on the dorsal surface of the hand before a scheduled surgery
Garcia et al., 2020 [22]	56	57	**Length:** Throughout the procedure **Induction:** Direct—eye-fixation **Maintenance:** Through headphones (Ericksonian and traditional conversational hypnosis) **Role of hypnotherapist**: Practitioners trained by the French Hypnosis Association	VAS	Pain during the whole procedure was significantly lower in the hypnosis group compared to the placebo group Hypnosis = 4.0 ± 2.2 Placebo = 5.5 ± 1.8 *p* < 0.001 OME: Intervention = 1.3 ± 1.3 Control = 3.6 ± 1.8	Atrial flutter ablation
Lee et al., 2019 [23]	8	9	**Length:** 35 min presurgical recording and postsurgical recording that needs to be listened to at least 24 h after surgery **Induction:** Indirect—audio with hypnotic recordings with background music **Role of hypnotherapist:** Psychologist experienced in pain management and clinical hypnosis	NRS	Pain in the hypnosis group was lower to a small extent compared to the control group. Baseline Hypnosis = 4.58 (1.86) Placebo = 4.60 (1.49) POH24 (before second treatment) Hypnosis = 2.28 (1.75) Placebo = 3.15 (2.39) POH24 (after second treatment) Hypnosis = 1.25 (0.88) Placebo = 2.38 (1.86) POD3 Hypnosis = 1.77 (0.83) Placebo = 2.59 (1.47) *p* = 0.316 OME: not reported	Total knee arthroplasty
Amraoui et al., 2018 [24]	77	71	**Length:** 15 min before surgery **Induction:** Indirect—personalized wording or nonverbal communication **Role of hypnotherapist:** Trained anesthesiologist with more than one year experience	VAS	No significant difference between the two groups. Baseline Hypnosis = 1.45 (2.05) Placebo = 1.44 (1.78) Post-anesthesia care unit, *p* = 0.77 Hypnosis = 1.00 (1.57) Placebo = 0.75 (1.19) Evening, *p* = 0.87 Hypnosis = 0.88 (1.45) Placebo = 0.88 (1.57) POD1, *p* = 0.88 Hypnosis = 0.87 (1.72) Placebo = 0.64 (1.13) 95% CI all OME: not reported	Breast cancer tumor removal
Akgul et al., 2016 [25]	22	22	**Length:** 30 min before the surgery **Induction:** Indirect—verbal **Role of hypnotherapist:** Anesthesiologist with experience in clinical hypnosis	VAS	Hypnosis group pain was lower compared with the control group Baseline, *p* = 0.31 Hypnosis = 0.05 ± 0.2 Placebo = 0.0 ± 00 POH2, *p* = 0.41 Hypnosis = 0.50 ± 0.9 Placebo = 0.73 ± 1.1 POH4, *p* = 0.39 Hypnosis = 0.91 ± 1.3 Placebo = 1.14 ± 1.2 POH6, *p* = 0.91 Hypnosis = 1.73 ± 1.1 Placebo = 1.77 ± 1.3 POH8, *p* = 0.001 Hypnosis = 1.64 ± 1.1 Placebo = 3.00 ± 1.3 POH10, *p* = 0.001 Hypnosis = 1.27 ± 1.1 Placebo = 2.95 ± 1.7 POH12, *p* = 0.002 Hypnosis = 1.73 ± 1.2 Placebo = 2.82 ± 0.9 POH24, *p* = 0.01 Hypnosis = 1.50 ± 1.1 Placebo = 2.27 ± 0.9 99%CI OME: Intervention = 4.9 (3.3) Control = 13.6 (2.7)	Coronary artery bypass grafting

**Table 2:** Mean and SD values of pain scores for acute pain randomized controlled studies.

	Placebo Treatment			Hypnosis Treatment			Total Number of Patients
Study	Mean	SD	Number of Patients	Mean	SD	Number of Patients	
**Overall total number of patients**							**888**
**Total number of patients for studies reporting NRS scores**							**583**
Nowak et al., 2020 [20]	5.33	2.24	194	4.33	2.24	191	385
Fusco et al., 2020 [21]	3.5	2.3	92	1.5	1.9	89	181
Lee et al., 2019 [23]	2.38	1.86	9	1.25	0,88	8	17
**Total number of patients for studies reporting VAS scores**							**305**
Garcia et al., 2020 [22]	5.5	1.8	57	4	2.2	56	113
Amraoui et al., 2018 [24]	0.64	1.13	71	0.87	1.72	77	148
Akgul et al., 2016 [25]	2.27	0.9	22	1.5	1.1	22	44

**Table 3:** Selected studies’ review for OME.

Study	OME, mg Mean (SD)	
	Intervention	Control
Nowak et al., 2020 [20]	4.0 (5.98)	6.43 (7.47)
Garcia et al., 2020 [22]	1.3 (1.3)	3.6 (1.8)
Akgul et al., 2016 [25]	4.9 (3.3)	13.6 (2.7)

**Table 4:** Selected studies’ reviews of chronic pain.

Study	Hypnosis Patients (n)	Control Patients (n)	Description of Hypnosis	Pain Measure	Outcomes in Mean (SD) [Range]	Chronic Pain Type
Tonye-Geoffroy et al., 2021 [26]	36	34	**Length:** 30 min at each visit POD0, POD7, POD21, POD30, POD42, POD56, POM3, POM6 **Induction:** Indirect—verbally **Role of hypnotherapist**: Qualified pain and anesthesiology nurse	VAS	No significant difference was observed between the control and intervention groups; Baseline Hypnosis = 5.63 (1.97) Placebo = 5.84 (2.23) POM3 Hypnosis = 3.38 (2.25) Placebo = 3.49 (2.03)	Chronic non-cancer nociceptive and neuropathic pain
Rizzo et al., 2018 [27]	50	50 (Pain education)	**Length:** Four sessions twice a week in a group of 1–7 participants. Also, home hypnosis workbook was given for self-practice **Induction:** Indirect—verbally **Role of hypnotherapist**: Physical therapist certified in hypnotherapy	NRS	No significant differences between the groups in average pain intensity Baseline Hypnosis = 6.63 (1.57) Placebo = 7.20 (1.61) POW2 Hypnosis = 4.4 (2.14) Placebo = 5.6 (2.21) POM3 Hypnosis = 4.4 (2.93) Placebo = 5.1 (2.48)	Chronic nonspecific lower back pain
Jensen et al., 2020 [28]	43	42	**Length:** Once a day **Induction:** Indirect—audio (adapted from an existing protocol utilized in a series of trials of self-hypnosis for chronic pain)	NRS	No significant differences between the groups Pretreatment Hypnosis = 4.47 (1.72) Placebo = 4.63 (1.82) POM3: Mean (95% CI) Hypnosis = 3.71 (2.802–4.618) Placebo = 3.69 (2.8–4.59)	Chronic lower back pain or chronic pain secondary to one of the following chronic conditions: multiple sclerosis, spinal cord injury, acquired amputation, or muscular dystrophy
Williams et al., 2022 [29]	110	110 (Pain education)	**Length:** 15–30 min, three interventions over 8–10 weeks **Induction:** Indirect—audio **Role of hypnotherapist**: Two-day in-person trained clinicians	NRS	No significant difference in pain in the hypnosis group in comparison with the control group Pretreatment: Mean (SD) Hypnosis = 5.7 (1.8) Placebo = 5.8 (1.6) POM3: Mean (95% CI) Hypnosis = 4.75 (4.344—5.156) Placebo = 5.32 (4.951–5.679)	Chronic pain
Bicego et al., 2021 [30]	52	47	**Length:** 20 min hetero-hypnosis exercise and daily self-hypnosis **Induction:** Direct—visual fixation and/or breathing attention focalization; five different exercises aimed to increase comfort and sleep quality and to decrease pain sensations **Role of hypnotherapist**: Clinical hypnosis specialists	NRS	No significant difference was observed between groups Baseline: Mean (SD) Hypnosis = 6.19 ± 1.45 Placebo = 5.74 ± 1.51 Posttreatment Hypnosis = 5.88 ± 1.62 Placebo = 5.42 ± 1.66 POM6 Hypnosis = 5.75 ± 1.91 Placebo = 5.51 ± 1.82 POM12 Hypnosis = 5.92 ± 1.86 Placebo = 5.57 ± 1.37	Chronic pain
Paredes et al., 2021 [31]	10	10	**Length:** 60 min four times a week **Induction:** Indirect—verbal communication **Role of hypnotherapist**: Certified clinicians	NRS	The differences in pain intensity from pre- to post-intervention were not significant between the groups. Baseline: Mean (SD) Hypnosis = 4.22 (1.99) Placebo = 4.27 (1.77) Posttreatment Hypnosis = 4.13 (1.09) Placebo = 3.81 (1.51)	Joint pain

**Table 5:** Mean and SD values of pain scores for chronic pain randomized controlled studies.

	Placebo Treatment			Hypnosis Treatment			Total Number of Patients
Study	Mean	SD	Number of Patients	Mean	SD	Number of Patients	
Tonye-Geoffroy et al., 2021 [26]	* 3.49	2.03	34	* 3.38	2.25	36	70
Rizzo et al., 2018 [27]	5.1	2.48	50	4.4	2.93	50	100
Jensen et al., 2020 [28]	3.69	2.87	42	3.71	2.99	43	86
Williams et al., 2022 [29]	5.32	1.93	110	4.75	2.15	110	220
Bicego et al., 2021 [30]	5.74	1.66	47	6.19	1.62	52	99
Paredes et al., 2021 [31]	3.81	1.51	10	4.13	1.09	10	20
Overall total number of patients							595

* VAS.

## Data Availability

Data are available on request.

## Supplementary Materials

The following supporting information can be downloaded at: https://www.mdpi.com/article/10.3390/jcm14134661/s1, Figure S1. Risk of bias assessment for acute pain studies [20,21,22,23,24,25]. Figure S2. Risk of bias assessment for chronic pain studies [26,27,28,29,30,31].

## Abbreviations

The following abbreviations are used in this manuscript:

BPI	brief pain inventory
CAM	complementary and alternative medicine
CI	confidence interval
NRS	numeric rating scale
OME	oral morphine equivalents
POD	post-operative day
POH	post-operative hour
POM	post-operative month
POW	post-operative week
REML	restricted maximum likelihood
RCT	randomized controlled trial
SD	standard deviation
TIS	tonic immobility state
VAS	visual analogue scale
VR	virtual reality
