# Supplementary material for: The Use of Medical Hypnosis to Prevent and Treat Acute and Chronic Pain: A Systematic Review and Meta-Analysis
Source: J Clin Med. 2025 Jul 1;14(13):4661. doi: 10.3390/jcm14134661 (PMC12250368; doi:10.3390/jcm14134661)
Supplement: Supplementary file 1 [file jcm-14-04661-s001.zip › jcm-3642545-supplementary.pdf]

# Risk of bias: Acute pain

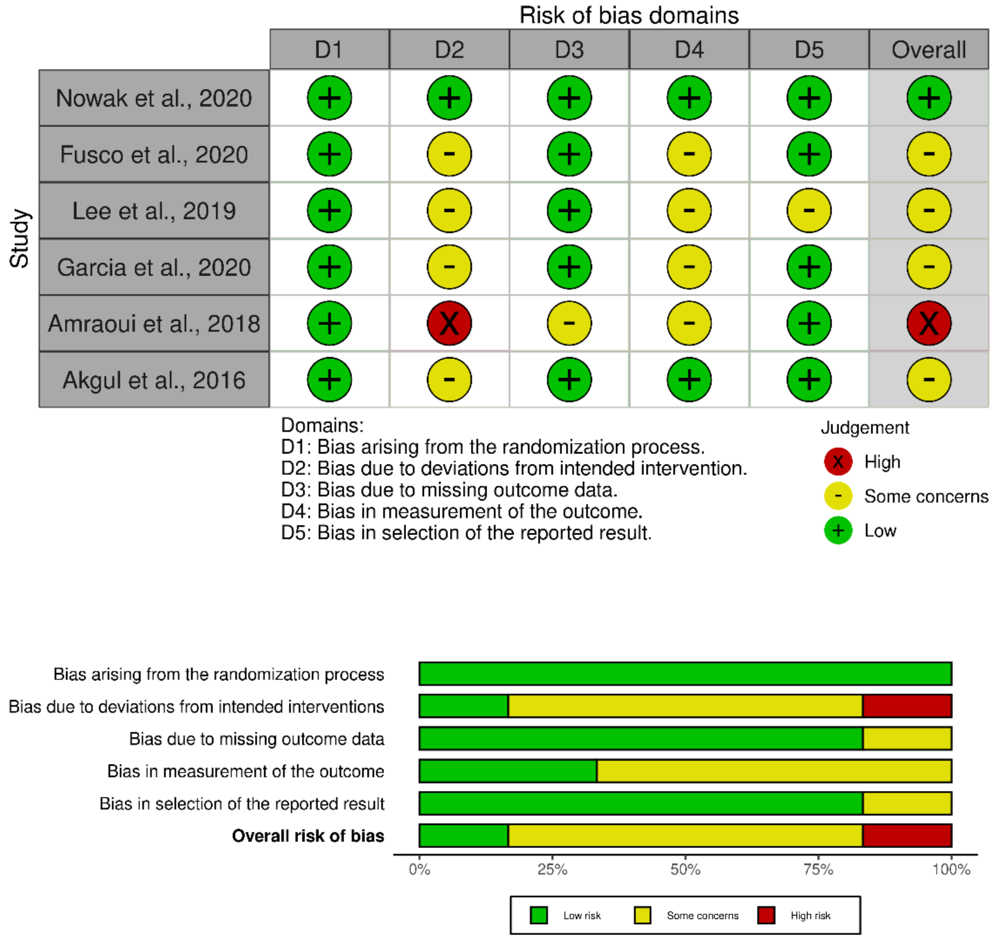


**Figure S1.** Risk of bias assessment for acute pain studies

# Risk of bias: Chronic pain

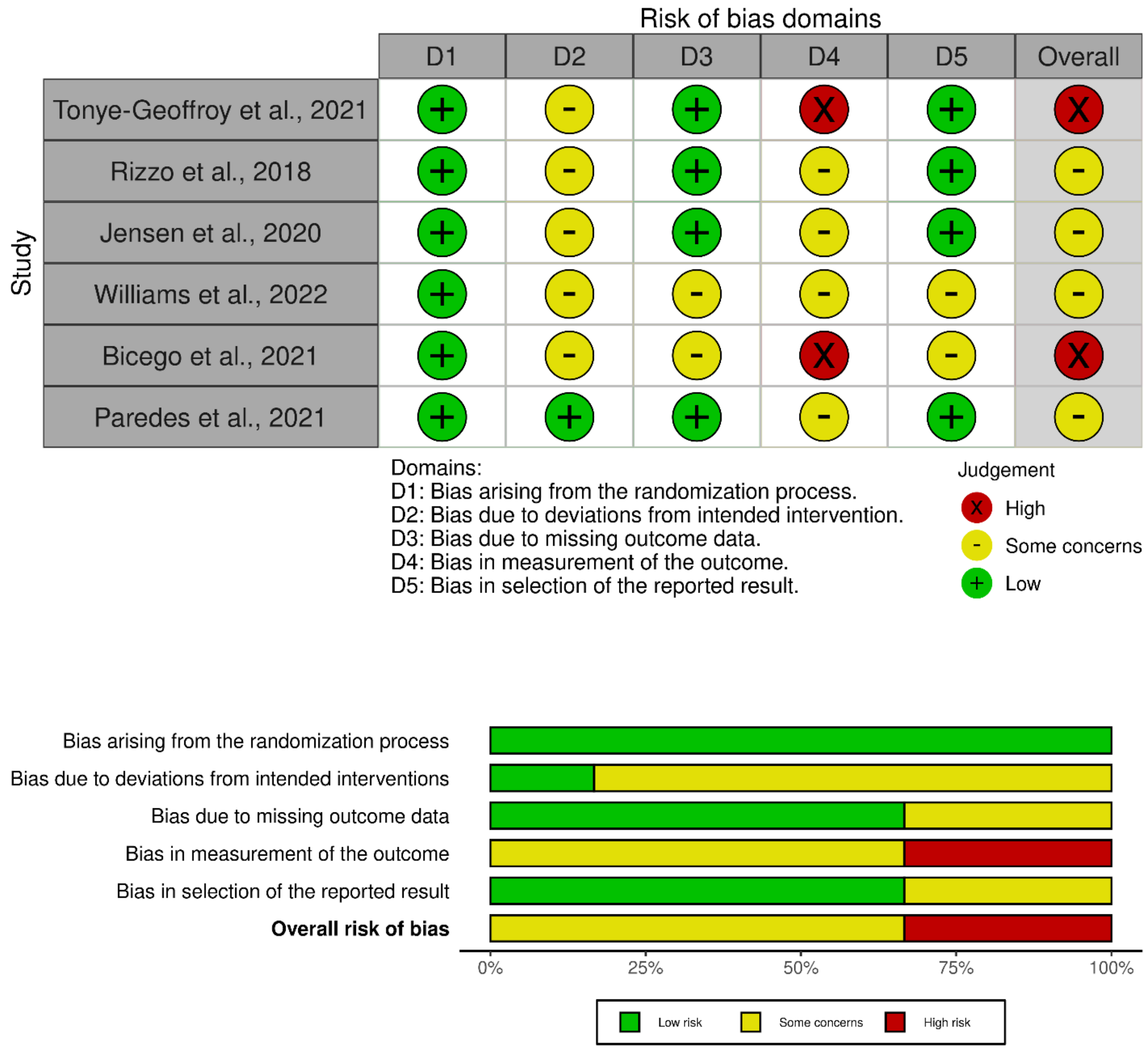


**Figure S2.** Risk of bias assessment for chronic pain studies
